# Tracking of Labelled Stem Cells Using Molecular MR Imaging in a Mouse Burn Model *in Vivo* as an Approach to Regenerative Medicine

**DOI:** 10.4236/ami.2021.111001

**Published:** 2021-01

**Authors:** Zeba Qadri, Valeria Righi, Shasha Li, A. Aria Tzika

**Affiliations:** 1 MGH NMR Surgical Laboratory, Center for Surgery, Innovation and Bioengineering, Department of Surgery, Massachusetts General and Shriners Burn Hospitals, Harvard Medical School, Boston, USA; 2 Athinoula A. Martinos Center of Biomedical Imaging, Department of Radiology, Massachusetts General Hospital, Harvard Medical School Charlestown, USA

**Keywords:** Burn Wounds, Cell Labeling, Cell Tracking, Cellular Imaging, Magnetic Resonance, Imaging (MRI), Molecular Imaging, Positive Contrast Imaging, Stem Cells

## Abstract

Therapies based on stem cell transplants offer significant potential in the field of regenerative medicine. Monitoring the fate of the transplanted stem cells in a timely manner is considered one of the main limitations for long-standing success of stem cell transplants. Imaging methods that visualize and track stem cells *in vivo* non-invasively in real time are helpful towards the development of successful cell transplantation techniques. Novel molecular imaging methods which are non-invasive particularly such as MRI have been of great recent interest. Hence, mouse models which are of clinical relevance have been studied by injecting contrast agents used for labelling cells such as super-paramagnetic iron-oxide (SPIO) nanoparticles for cellular imaging. The MR techniques which can be used to generate positive contrast images have been of much relevance recently for tracking of the labelled cells. Particularly when the off-resonance region in the vicinity of the labeled cells is selectively excited while suppressing the signals from the non-labeled regions by the method of spectral dephasing. Thus, tracking of magnetically labelled cells employing positive contrast *in vivo* MR imaging methods in a burn mouse model in a non-invasive way has been the scope of this study. The consequences have direct implications for monitoring labeled stem cells at some stage in wound healing. We suggest that our approach can be used in clinical trials in molecular and regenerative medicine.

## 1. Introduction

Stem cells (SCs) are a group of undifferentiated pluripotent cells characterized by the aid of the capability to undergo self-renewal as well as differentiation into various other tissue types which in turn depends on what type of tissues they have been derived from [1]. SCs can be of embryonal origin in which case the embryonal SCs are derived from embryonal tissue. On the other hand SCs can also be of adult origin, and can be derived from adult germ cells [2]. Adult SCs are derived from adult tissues of different organs consisting of intestines and bone marrow, which are taken into consideration to have excessive turnover [2].

Though in general SCs have been implicated in the facilitation of wound healing, nevertheless the satisfaction is yet to be found concerning the usage of stem cell therapy [3]. Substantial studies using SCs are being carried out with promising effects in various fields ranging from wound healing, hematologic diseases, oncologic diseases, organ transplants and so forth. The potential clinical application of different types of SCs has been reported for specific varieties of wounds in the field of wound healing [4] [5] [6] with a special interest in burning wounds [3]. The use of mesenchymal bone marrow derived stem cells (BMSCs) for burn wound recovery in rats, as stated through Shumakov *et al*. [7] had been the first to use stem cells in the area of burn wound healing. Rasulov *et al*. [8] had been the first who applied SCs onto the burn surface by using BMSCs in humans and also showed that the application of SCs results in a quicker healing of wounds [8]. Some other observations on rats also supported the fact that stem cells are a superior choice in the healing of burn wounds [9]. Further it is added that use of mesenchymal SCs on burn wounds improves formation of blood vessels and decreases unusual growth of cells [9].

So far research has highlighted the position of SCs in the process of wound recuperation. Burn wound recovery has also been a subject of additional focus. However, SC application to burn wounds comprises of various methods [9] [10] [11] [12], These strategies may include local or IV injections or topical application. Due to properties like self-renewal and migration which are of utmost importance for wound healing treatment options based on SCs keep top notch guarantees in regenerative medicinal drug [7]-[12].

In order to understand the utility of SC technology in the treatment of burn wounds their *in vivo* tracking over time becomes crucial. Also, to understand the repair work tracking and quantification of SCs is critical. Labeling of cells is essential to tune their migration and differentiation via imaging post-transplantation. Cells can be labeled by means of direct or oblique techniques. In the direct methods there are no modifications of the genetic material of the cell. On the other hand, the oblique methods of cell labeling require alterations of the genetic material. This is carried out by the introduction of a reporter gene into the genetic material which is a more complicated process. Hence, direct labeling methods are preferred and are more common. Direct labeling is done by delivering a labeling agent into the cell. This is followed by the transplantation of the labeled cells. These transplanted cells are then imaged indirectly [13]. Labeling agents can be classified on the basis of imaging modalities to be used [14] [15]. These include fluorochromes for optical fluorescence imaging, radioactive nuclides for positron emission tomography (PET) or single photon emission computed tomography (SPECT), paramagnetic and super-paramagnetic iron oxide (SPIO) particles for MRI [16] [17] [18]. Of all the listed labeling agents the MR contrast agents have some advantages because they provide simple, faster, effective and long-term tracking of the cells involved in the healing process. Because of these properties the MR labeling agents [19]. Considering the extremely hard work being carried out to improve cellular imaging techniques, MRI has the advantage of better resolution over optical imaging. MRI also has an added advantage of anatomical information of high spatial resolution over PET [20].

MRI being one among the sophisticated and non-invasive techniques that visualize the internal structures of the body in healthy as well as diseased conditions. It provides better resolution and contrast for soft tissues. It doesn’t involve any harmful radiations and exhibits low toxicity. It has as a result been used as a diagnostic device, with a wide range of medical applications, for over three decades now [21] [22] [23]. MRI is a spectroscopic technique which depends on the quantum mechanical spin properties of the hydrogen nuclei which results in a magnetic moment. Under the influence of a strong applied magnetic field the magnetic moments of those nuclei align with the applied magnetic field. Upon the application of an RF pulse the nuclei get excited to the higher spin energy state. This is followed by relaxation to the lower spin energy state. During the relaxation to the equilibrium state the absorbed energy is again released in the form of an RF signal which is detected and subsequently processed resulting in the generation of a cross-sectional anatomical image.

The idea at the back of the anatomical images furnished with the aid of MRI is T_1_ (water spin-lattice or longitudinal) and T_2_ (water spin-spin or transverse) ^1^H relaxation processes of water found in soft tissues [24] [25]. Many times the endogenous MR differences due to inherent relaxation variations among various sorts of tissues may not be enough to develop an image contrast. For this reason, an exogenous contrast agent (CA) is regularly utilized in MRI to provide additional assessment to differentiate a target tissue from its surroundings. With the aid of the use of MRI contrast agents the assessment of images and subsequently the visibility of specific anatomical structures may be progressed. While employing Magnetic Resonance Imaging, cells are labeled with an appropriate contrast agent. Further research in this area made the use of these contrast agents feasible for labeling of specific cell types [26]-[36]. Regenerative medicine research field has been benefited using MRI for *in vivo* imaging and tracking of engrafted SCs. MRI has also proved to be useful to observe the physiological responses in a safe and non-invasive way.

The metal ions present in the MR contrast agents exhibit characteristic relaxation enhancements. These agents catalytically shorten the relaxation times (T_1_ & T_2_) of nearby tissue water protons to various degrees relying on their nature, making them discernible on post-contrast MRI. For cell labeling and cell tracking the MR contrast agents used are of the following types: 1) paramagnetic agents [26] [27] [28]; 2) super paramagnetic agents [29]; and 3) contrast agents that contain nuclei other than hydrogen [30]–[36].

Paramagnetic contrast agents create a magnetic moment which decreases the longitudinal relaxation time (T_1_) and the transverse relaxation time (T_2_) of the surrounding water protons through more or less the same quantity. Those contrast agents which exhibit a positive contrast are known as T1 agents. A positive contrast refers to a bright image quality. The signal thus obtained is referred to as T1 weighted. But the major disadvantage of paramagnetic agents in cell tracking is that the signal drops off with decreasing number of labeled cells. On the other hand, super-paramagnetic contrast agents such as SPIO nanoparticles lowers the transverse relaxation time (T2) to a far greater extent and are known as negative contrast agents which produce darker image quality [37].

*In vivo* MR imaging of SPIO labeled cells is generally carried out using gradient- or spin-echo sequences for T_2_- or ${\text{T}}_{2}^{*}$ -weighted negative-contrast [38] [39]. Negative contrast methods function by imaging the accumulated SPIO nanoparticles via loss of signal intensity due to strong ${\text{T}}_{2}^{*}$ dephasing which is induced by the local presence of super-paramagnetic nanoparticles. This makes quantification of signal loss difficult especially in regions of high concentrations of SPIO due to low signal to noise ratio (SNR) as a result of dephasing. To avoid the signal loss positive contrast MR imaging is a better option to negative contrast. This is done by exploiting the chemical shifts induced by SPIO in neighboring water molecules. There are various strategies for the generation of a positive contrast. These methods make use of specially designed pulses to bring about the refocusing of the water signals in the vicinity of the SPIO particles [40]. The responsiveness of SPIO particles towards MRI is much stronger as compared to the paramagnetic agents. These SPIO particles tend to induce a local field inhomogeneity, which gives rise to a dipolar magnetic field large enough to cause signal spin-spin dephasing [41]. This gives rise to a negative contrast on T2-weighted MRI and with high local concentrations of the superparamagnetic contrast agents may produce a complete signal void. This situation could be overcome if the imaging contrast is provided by transverse (T_2ρ_) relaxation in the rotating frame [42] [43] which could be achieved through the application of a spin-lock RF (radio-frequency) pulse, which together with the static magnetic field results in an effective field. This situation may be conquered if the imaging evaluation is carried out via Off-Resonance Imaging (ORI) (44) therefore developing a positive (bright) contrast.

**Figure 1.**
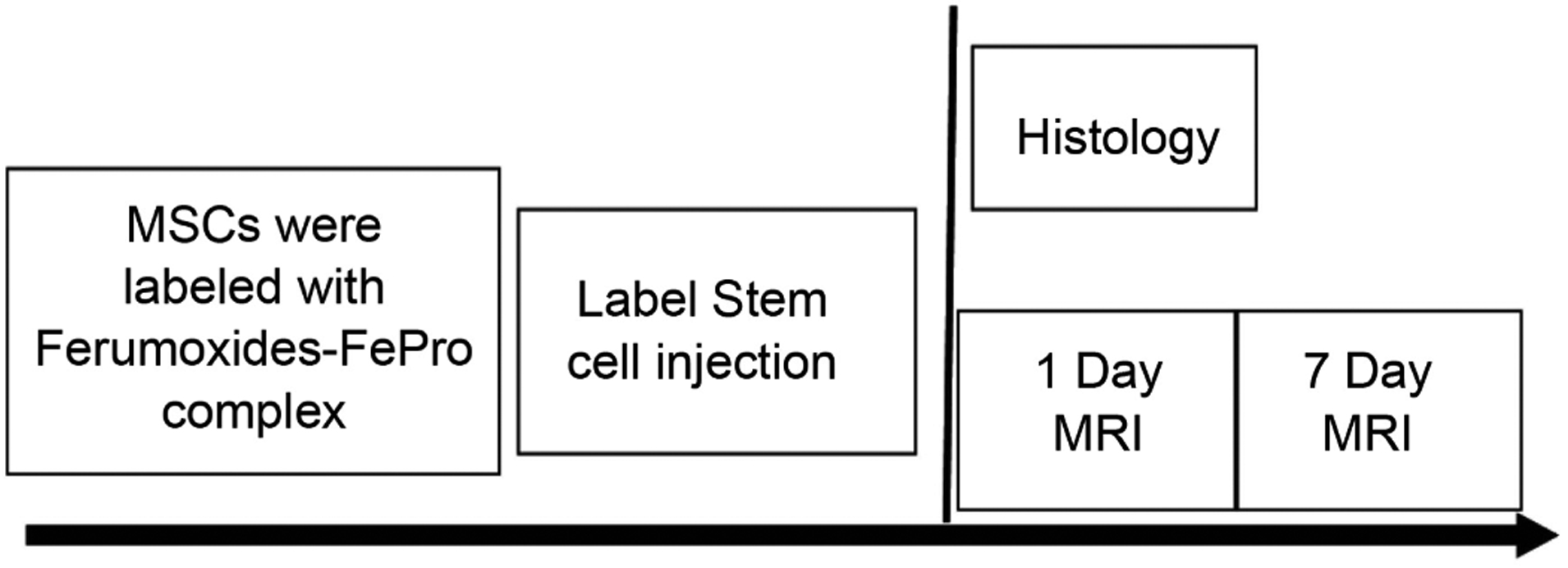
Schematic of the experimental protocol illustrating the contrast agent (Fe-Pro Complex) ingestion into the MSCs for labelling followed by labelled stem cell injection into the mice, followed by the magnetic resonance time experiment and the last step of histochemical staining of the labelled cells.

Non-invasive techniques for molecular imaging involving novel strategies making use of SPIO nanoparticles have been of recent interest [45] [46], but there are several techniques which help in generating tremendous positive contrast of the magnetically labelled cells. This is brought into effect by selective spectral excitation of an off-resonance region in the vicinity of the labelled cells [40]. During this process the signals from the non-labeled regions are suppressed [47] [48]. The tracking of SCs labelled with SPIO in a burn mouse model will be addressed here in particular employing non-invasive *in vivo* MR methods for generating positive contrast imaging (Schematic is shown in Figure 1).

## 2. Materials and Methods

### Mice:

Five GFP+ mice (28 – 30 g) were used to carry out the studies. An i.p. injection of 40 mg/kg pentobarbital sodium was injected into the hind limb the mice for anesthetizing it. The mice were subjected to scald injury of non-lethal nature, representing merely 3% – 5% of the total body surface area, by immersion in 90°C water for a time period of about 9 sec [49] [50]. According to requirements s.c. buprenorphine at 0.05 – 0.1 mg/kg, was provided as an analgesic.

### Isolation and preparation of stem cells (BMSCs):

Mesenchymal stem cells (multipotent stem cells which can easily differentiate to keratinocytes and epidermis) were isolated from bone marrow and confirmed their characteristics with flow cytometry. GFP+ mesenchymal stem cell lines were obtained from Tulane University. Cells were proliferated under conditions that keeps them proliferating without changing their surface markers (and state of differentiation). The isolated cells showed MSC markers and could be differentiated from different cell lines *in vitro*.

### Labelling of BMSCs, histology and optimization:

The GFP+ multipotent cell line (BMSCs) isolated from bone marrow of GFP+ mice were labeled with the help of Ferumoxide-protamine sulphate (Fe-Pro) complexes (around 10 μg intracellular iron) which is a type of synthetic SPIO. These magnetically labelled BMSCs were then injected into the mice to serve the purpose of a molecular imaging MRI contrast agent. Hydrochloric acid and Potassium ferrocyanide were used in combination with Spectrophotometric methods for determining the “Mean Intracellular Iron” in the labelled cells [51]. After 48 to 72 hours of the thermal injury about 3 million MSCs were injected into the mice intravenously. These cells could be tracked with MRI and fluorescent microscope and we could show the migration of stem cells to the area of interest.

### Imaging:

**Figure 2.**
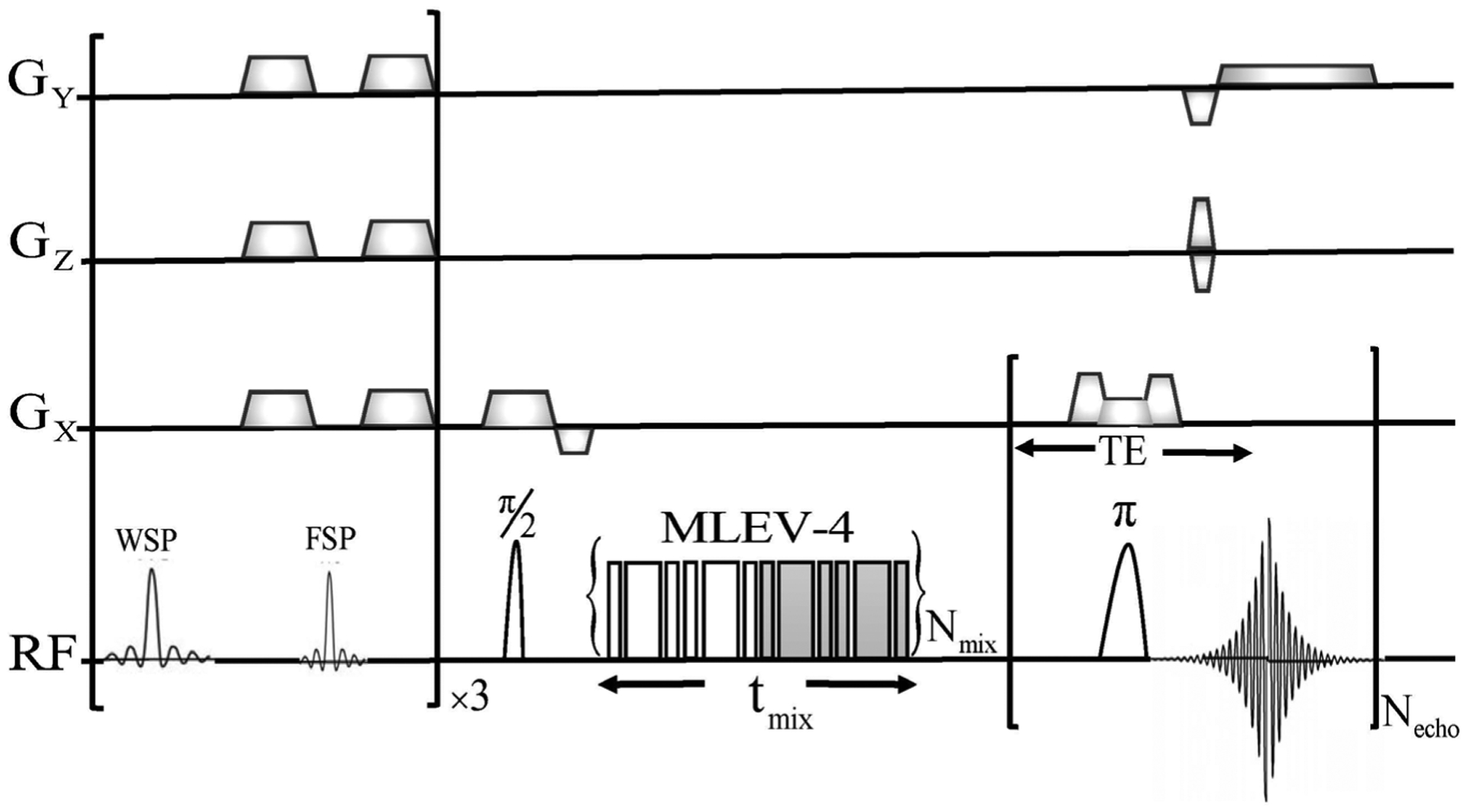
Schematic for the Off-Resonance Imaging (ORI) and ORI-transverse (*T*_2ρ_) relaxation in the rotating frame positive contrast pulse sequence. The sequence consists of a frequency-selective bandwidth for water- and fat-suppression pulses (WSP, FSP). A RARE MRI sequence with two echoes (Necho = 2) was employed for acquiring images. A spin-locking pulse block is inserted between the 900 excitation and 1800 inversion RF pulses a spin-locking pulse block is employed for establishing ORI-T2ρ. The MLEV-4 mixing scheme, with the constant adiabaticity HS4 1800 pulses are employed for spin-locking. In order to increment the mixing time the number of MLEV-4 blocks (Nmix) are repeated the desired number of times. A zero mixing time (Nmix = 0) corresponds to the ORI sequence.

After 48 hours of the IV injection comprising of the iron labelled MSCs two mice were scanned in a 4.7 T horizontal bore magnet (20 cm bore diameter, Magnex Scientific, using a Bruker Avance console). While imaging the mice both positive and negative contrasts MR images were acquired. The Positive contrast MR method was employed using an off-resonant imaging (ORI) technique [44] implemented in a RARE (Rapid Imaging with Refocused Echoes) [52] sequence with RARE acceleration factor two. The ORI-T_2ρ_ technique (schematic shown in Figure 2, as adapted from Andronesi *et al*. [53] was chosen to implement in a RARE sequence. An acceleration factor of two was achieved via the insertion of an MLEV-4 block. The HS4 adiabatic pulses constitute the MLEV-4 block for attaining relaxation in the rotating frame [52]. The optimization of the RF amplitude and that of the adiabatic pulses in the ORI-T2ρ for setting the mixing time had been done for *in vivo* imaging of the mice [53]. Ten-lobed sinc pulses for selective water (400 Hz bandwidth) and fat (800 Hz bandwidth) suppression were applied. This was followed by decohering the transverse magnetization through spoiling gradients. Following the water and fat suppression pulses as mentioned a spin-echo imaging sequence was applied. A negative contrast was achieved with a series of FLASH (Fast Low-Angle SHot) [54] images. Increased echo-time was used to achieve negative contrast. For the ${\text{T}}_{2}^{*}$ weighting typical values α = 15°, TR = 500 ms, TE = 4, 6, 8, 12, 14 ms (Figure 2) were employed. For each mouse the total MRI scan time was 2.5 h approximately. Two weeks after imaging the mice were euthanized for performing histology studies. Prussian blue staining of iron labelled cells was done for histochemical determination. One week later after the first analysis the same experiment was repeated for the second mouse.

## 3. Results

**Figure 3.**
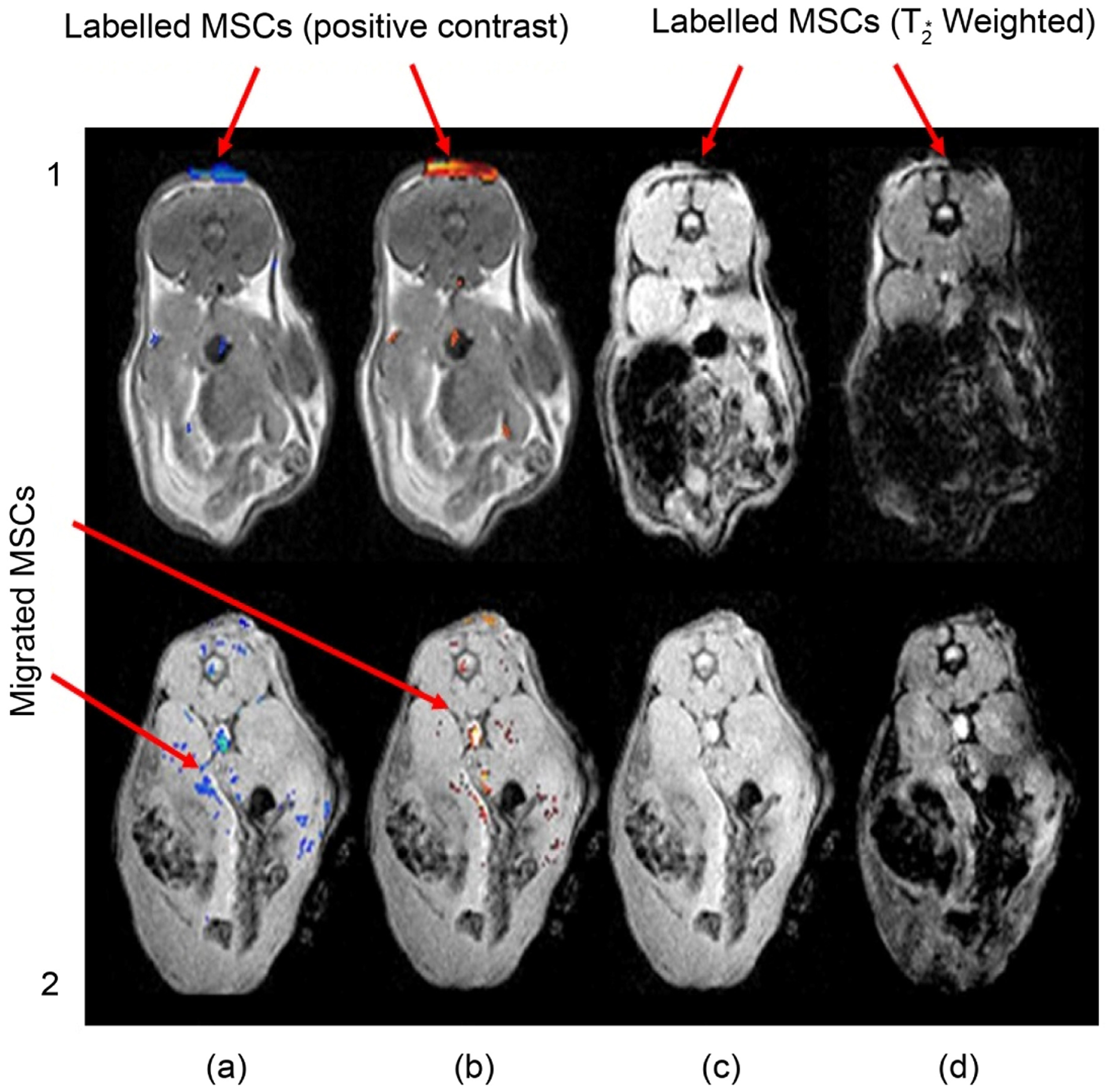
*In vivo* imaging of labeled stem cells in a mouse burn model, 1) after 24 h and 2) one week following injection. (a) Off-Resonance Imaging (ORI), (positive contrast); (b) ORI-*T*_2ρ_ (positive contrast); (c) FLASH (TE = 4 msec) (negative contrast), (d) FLASH (TE = 14 msec) (negative contrast). Top-most region of every image in panel (1) shows accumulated SPIO labelled MSCs. In panel (2) the labelled MSCs show migration.

Ten axial slices of 1 mm thickness, 1.5 mm gap, 3 × 3 cm FOV, 128 × 128 matrix size and 8 averages were acquired during the MRI scan. The *in vivo* MR images of the labeled SCs in the burn vicinity are shown in Figure 3. For the demonstrative slices positive-contrast images with pseudocolor were obtained which have been shown to superimpose on the proton-density weighted anatomical images from the imaged mice. Images resulting from ORI-T2ρ as well as ORI schemes were converted to SNR images. The threshold SNR level for these images was kept the same so that it could be used as a foundation for comparison for both the protocols employed for MRI. In both the mice similar slices were taken from the same anatomical location. MR images were taken 24 h after the IV SC injection as shown in Figure 3, panel 1 and those of a week later are shown in Figure 3, panel 2.

The positive-contrast images were thresholded to signal >3 in dimensionless units of SNR and were superimposed on a T1-weighted FLASH image and presented in pseudo color. In Figure 3 from left to right, images are: (a) ORI, (b) ORI-T_2ρ_, (c) FLASH (TE = 4 msec), (d) FLASH (TE = 14 msec). Image processing (Figure 3) was carried out by drawing around the area of the burn for selecting regions of interest (ROI) and integrating the total (thresholded) signal intensity within each ROI. The histology for the injected SPIO labeled MSCs (blue) was done manually by counting the number of stained (with Prussian blue) and unstained cells under a microscope. he effectiveness of the labeling methods was found to be greater than 95%. *In vivo* MRI tracking of these labeled SCs was done non-invasively with a high degree of sensitivity and specificity. Our results thus showed the efficient labeling of MSCs with ferumoxides-protamine sulfate complexes.

## 4. Discussion

In the current effort we geared in the direction of refining the specificity for SPIO detection. The possible applications of SCs in the process of treatment of burn injuries and how the healing progress can be monitored have been presented in this study. In numerous cases the approaches above are not always specific to SPIO and either negative- or positive- contrast like sections appear in places where it is not anticipated.

**Figure 4.**
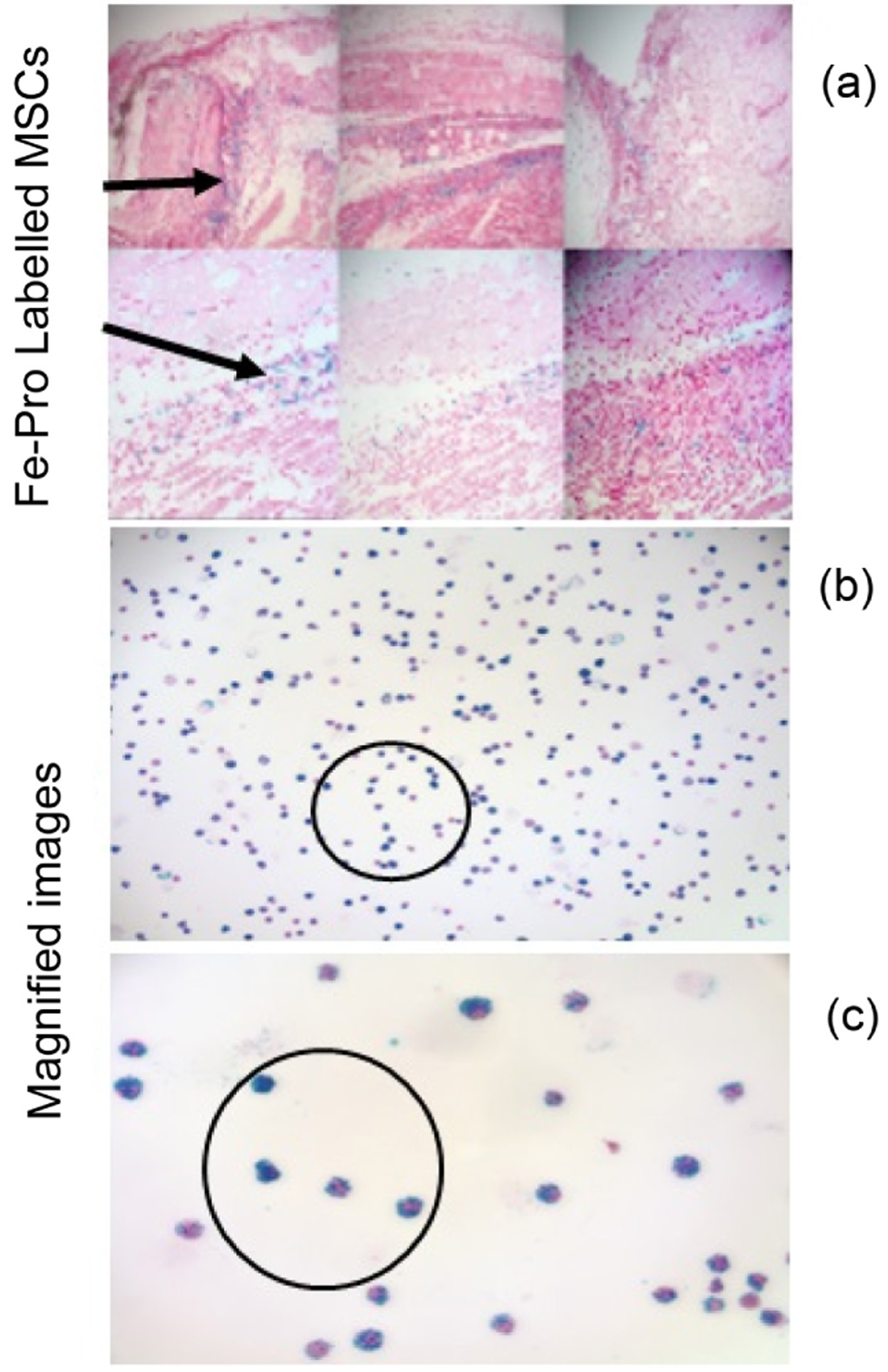
Histology, Prussian blue staining for Iron labeled stem cells. (a) The blue dots correspond to the Fe-Pro labelled MSCs. Panels (b) and (c) show magnified images of the same with a closer view of the labelled cells.

The MR images evidently showing the relocation and gathering of the iron labelled SCs to the area of the burn injury (Figure 3, panels 1(a) & 1(b)) constitutes the major finding of this study. The relocation of the iron labeled SCs was confirmed with the aid of histology staining using Prussian blue as shown in Figure 4. The studies shown corroborate Dr. Alan Fischman’s findings [55] but his work makes use of radioactivity. Ours is non-irradiating, the cells may well be traced non-invasively with high sensitivity and specificity *in vivo*, which is a primary advantage of MRI, over nuclear medicine methods [56] [57]. When MRI was performed after one week of injection of iron labeled MSCs to check if they could still be found in the burn area, positive contrast method did not reveal any which was then confirmed by ${\text{T}}_{2}^{*}$ weighted MR images with varying echo times.

We posit that the imaging of magnetic nanoparticles with positive contrast techniques, such as ORI, has the potential to turn out to be an exceedingly beneficial tool in cell tracking and molecular imaging. We have used the current method before to image burn and our findings here extend our previous findings (Andronesi *et al*.) [53]. Magnetically labeled SCs with a high concentration of nanoparticles [9], and accumulated endogenous ferritin [8], have already been successfully imaged with these strategies at 1.5 T. Imaging at 1.5 T lacks detail due to low sensitivity and specificity. However, ORI have been used for different field strengths. These studies play an important role for assessing the prospect of ORI in imaging the iron labelled regions at high fields in order to attain higher resolution. For sensing lower and diffuse concentrations of iron also these studies are useful. The analytic precision of ORI shows a non-linear response with the increase in the concentration of the nanoparticle [44]. Demonstration of a positive contrast requires the design of a special spectral-spatial pulse [58]. The investigative precision of the method employing an ORI scheme employing narrowband frequency-selective pulses to suppress the signals arising from water and fat is indicated besides a broadband spin-echo segment to image the excited spins in the locale of the suppressed water [59].

Enhancing the contrast of images of the engrafted stem cell is critical for the improvement of wound healing [60]. Thus, non-invasive MR visualization techniques for stem cells *in vivo* like the one demonstrated here has the ability to significantly enhance our understanding underlying mechanisms that would lead to efficacious engraftment consequences [61]. Formerly various tissues which were of interest were made to be transplanted with iron labeled cells were imaged with the help if MRI up to weeks [1]. Here, we performed the experiment with two methods, an ORI and an ORI-*T*_2ρ_ method [53]. The ORI-*T*_2ρ_ scheme offers some advantages over the ORI scheme which includes the adjustment of the B_1_ field in addition to the mixing time of the spinlock. This might permit a refinement of the contrast levels to specific concentration ranges [53]. Off-resonance MRI approaches produce positive-contrast where, a spectrally selective RF pulse is employed for exciting the off-resonance water signals [40] at the frequency shift induced by the iron particles and refocused such that elimination of the on-resonance background signal takes place at large. The ORI-*T*_2ρ_ method proved to have slightly higher sensitivity. It has potential in the sense that it future work may be benefited from this work. This is the first demonstration where ORI-*T*_2ρ_ method was used to tract SCs in the burn wounds and showed that SCs moved to populate and heal the burn wound (Figure 3). The beauty of our research is that we have the ability to follow the cells *in vivo*. Theoretically after every division the amount of iron decreases in cells so the MRI Signal to noise will decrease. It is of major importance that the cell labeling employed FDA (US Food and Drug Administration) approved reagent will allow this procedure to be performed in human subjects in the near future.

## 5. Conclusion

In conclusion, we have demonstrated the feasibility of employing positive-contrast method which combines off-resonance positive-contrast imaging with *T*_2ρ_ relaxation to image labeled stem cells *in vivo*, in a clinically relevant burn mouse model. This possibly will prove to be of greater efficacy than the ORI method. The outcomes might have undeviating implications for assessment and longitudinal tracking of labeled stem cells during wound healing. We suggest that our approach can be used in clinical trials in molecular and regenerative medicine.
